# Supplementary material for: L-phenylalanine Increased Gut Hormone Secretion through Calcium-Sensing Receptor in the Porcine Duodenum
Source: Animals (Basel). 2019 Jul 24;9(8):476. doi: 10.3390/ani9080476 (PMC6719913; doi:10.3390/ani9080476)
Supplement: Supplementary file 1 [file animals-09-00476-s001.pdf]

animals

Supplementary File: L-phenylalanine Increased Gut Hormone Secretion through Calcium-Sensing Receptor in the Porcine Duodenum

Jiangyin Feng, Cuicui Kang, Chao Wang, Liren Ding, Weiyun Zhu and Suqin Hang *

Table S1. Primers used in present study.

Targets	Gene Bank No.	Primers Sequences
CaSR	NM_001278748.1	Sense: 5′-CGGGGGACTCTTTCCTATTC-3′<br>Anti-sence: 5′-GCTGGGCTGCTGTTTATTTC-3′
CCK	NM_214237.2	Sense: 5′-CTTGTGCCTGTGTGTGCTG-3′<br>Anti-sence: 5′-AGATACTCGGCCAGAAGGTG-3′
GIP	NM_001287408.1	Sence: 5′-TTCTGTCTGCTGCTGGTGTC-3′<br>Anti-sence: 5′-CTCCCTCTGGGTGATGTTGT-3′
GAPDH	NM_001206359.1	Sense: 5′-ATGGTGAAGGTCGGAGTGAAC-3′<br>Anti-sence: 5′-CTCGCTCCTGGAAGATGGT-3′
β-actin	DQ178122.1	Sense: 5′-TCTGGCACCACACCTTCTACA-3′<br>Anti-sence: 5′-ATCTGGGTCATCTTCTCACGG-3′

CaSR: calcium-sensing receptor; CCK: cholecystokinin; GIP: glucose-dependent insulinotropic peptide; GAPDH: glyceraldehyde phosphate dehydrogenase.

© 2019 by the authors. Licensee MDPI, Basel, Switzerland. This article is an open access article distributed under the terms and conditions of the Creative Commons Attribution (CC BY) license (http://creativecommons.org/licenses/by/4.0/).
